# Association Between Globulin and Diabetic Nephropathy in Type2 Diabetes Mellitus Patients: A Cross-Sectional Study

**DOI:** 10.3389/fendo.2022.890273

**Published:** 2022-07-08

**Authors:** Jian Wang, Fang Liu, Rongzhen Kong, Xiuxia Han

**Affiliations:** Renal Department, Dezhou People’s Hospital, Dezhou, China

**Keywords:** globulin, logistic regression, curve fitting, type 2 diabetes mellitus, diabetic nephropathy

## Abstract

**Background:**

With the development of economy, the living standard of people all over the world has been greatly improved, and the incidence of diabetes is also increasing. Many people with diabetes also develop other complications that reduce their quality of life. Diabetic nephropathy is a common complication of type2 diabetes. Understanding the related factors of diabetic nephropathy is greatly significant to control the occurrence of diabetic nephropathy and improve patient’s life quality.

**Data and Methods:**

Data were collected from 2009 to 2018 in NHANES. Curve fitting graph was performed to investigate the association between globulin (GLB) and diabetic nephropathy(DN). Four logistic regression models were conducted to control the potential confounding factors. Subgroup analysis was carried out to assess the stability of results.

**Results:**

GLB was positively correlated with the occurrence of DN after controlling for potential confounders. Higher GLB was associated with an increased risk of diabetic nephropathy [odds ratio(OR), 1.10; 95% confidence interval (CI), 1.07-1.13, *P* < 0.001].

**Conclusions:**

In this cross-sectional study, GLB was significant positively correlated with the occurrence of DN in patients with type2 diabetes mellitus.

## Introduction

Diabetes mellitus, characterized by chronic hyperglycemia, is a global metabolic disease caused by spontaneous metabolic disorders (1, 2). Recently, the diabetes incidence has increased significantly (3). It is estimated that by 2,045, there will be 642 million people worldwide living with diabetes, according to a report released by the International Diabetes Federation (4). Diabetes, is one of the most common chronic diseases, which brings great pressure to patients and medical system (5), especially to those patients with diabetes complications, such as nephropathy (6). The occurrence of diabetic nephropathy can shorten lifespan of diabetics. A cohort study of diabetes by Livingstone SJ et al. revealed that the life expectancy of patients with diabetic nephropathy was shorter than that of patients without diabetic nephropathy, which may be one of the main reasons for the decline of diabetic patients’ life expectancy (7). In 2016, Miller RG et al. revealed that the occurrence of diabetic nephropathy may lead to an increased risk of cardiovascular disease in diabetic patients albeit in early stages of diabetic nephropathy (8). Simultaneously, some related studies have indicated that people with diabetic nephropathy are at higher risk of death after contracting COVID-19 (9). Therefore, a comprehensive understanding of diabetic nephropathy and its related factors may contribute to the prevention and control of diabetic nephropathy, thus improve the prognosis and quality of life of diabetic patients.

Globulin is a serum protein existing in the human body, which is also known as immunoglobulin because of its immune function. Globulin is mainly composed of α 1, α 2, β and γ (10). In previous studies, globulin was considered as a sensitive indicator of liver function damage (11), especially in cases of severe liver disease. Globulin is also a reliable biomarker that is readily available in the basal metabolome. However, the relationship between globulin and diabetic nephropathy in diabetic patients has not been elucidated yet.

Consequently, we aim to use the real world public database of NHANES screening diabetic participants in its 5 cycles (2009-2018), investigating the relationship between GLB and diabetic nephropathy and assess its clinical value.

## Methods

### Participants Selection and Study Grouping

The dataset for five ten-year periods from 2009 to 2018 were downloaded from the NHANES official websiteand weighted. We divided all participants into diabetic participants and non-diabetic participants, according to the commonly used international diagnostic criteria for diabetes. All patients with diabetes were included in this study. Meanwhile, we excluded people without GLP, ACR and other relevant data. This study is a cross-sectional study. Participants with ACR ≥ 30ug/mg were defined as diabetic nephropathy and those with ACR < 30ug/mg as non-diabetic nephropathy.

### Data Collection

The basic information of the population was collected by trained professionals, and all experimental measurement data were strictly performed in the whole process by professionals in accordance with the technical standards published on the official website of NHANES. All data and experimental methods can be downloaded from the NHANES website. The experiments were carried out in a laboratory in Minnesota.

Demographics data (sex, age, race/nationality, etc.), anthropometric measurements (height, weight and waistline, etc.), health-related behaviors (smoking and drinking, etc.), biochemical tests (high density lipoprotein, fasting blood glucose, albumin, globulin, total cholesterol, uric acid, etc.) were selected (12). Then all units were quantified in terms of international standard units. Globulin is a group of proteins that transport various substances in the blood, and are involved in various defense mechanisms in the body (13). Serum globulin is the measure of dividing the total protein by the albumin.

### Evaluation Criteria

#### Diagnosis of Diabetes Mellitus

The diagnostic criteria of diabetes were formulated by referring to international and previous research literatures (14). The criteria is: taking diabetes drugs, fasting blood glucose ≥ 7.0mmol/L or glycosylated hemoglobin ≥ 6.5mmol/L. The measured blood glucose values are rounded to three decimal places and converted from mg/dL to mmol/L.

#### Measurement of BMI

The body mass index (BMI) is the measure of dividing the weight (kg) by the square of the height (m^2^) (15). According to World Health Organization standards, BMI of 18.5 kg/m^2^ to 24.9 kg/m^2^ is normal, BMI of 25.0 to 29.9 kg/m^2^is overweight, and BMI ≥ 30.0kg/m^2^ is obese (16).

#### Diagnosis of Hypertension

Participants were assessed for high blood pressure using the average of three or two measurementsof all participants’ blood pressure, if only once was taken directly into the study, and assessed whether they were taking hypertension drugs. Subjects with systolic blood pressure ≥ 140mmHg and/or diastolic blood pressure ≥ 90mmHg were identified as hypertension (17).

#### Determination of Alcohol Consumption and Smoking Determination

We defined participants’ drinking status based on clinical experience combined with previous relevant studies, and finally divided alcohol consumption into two levels (18, 19). Non-drinker, a person who have no more than 12 drinks a year, more than 12 are considered drinkers. There are also two levels of smoking. According to previous research combined with data analysis, people with no more than 100 cigarettes in their lifetime or those who do not smoke into a group and are defined as non-smokers, because the number of non-smokers in the data was too small. Participants who are current smokers or had smoked more than 100 cigarettes are smokers (20).

#### Covariable Screening

Here, we filter the covariates according to the following rules.

(1) demographic data;

(2) factors that may affect diabetic nephropathy reported in previous literature;

(3) the introduction of variation leads to the change of regression coefficient of the basic model by more than 10%;

(4) based on our clinical experience.

Demographics include age, sex and race. Biochemical indicators include GGT, BUN, Hba1c, ALT, AST, UA, TP, ALB. Other covariates include height, weight, BMI, blood pressure, etc. Height and weight were measured by rangefinder and electronic scale (21). BMI and blood pressure were described above.

#### Statistical Methods

Using R language (version 4.10) and Free Statistics analysis platform for statistical analysis, and bilateral P< 0.05 was considered statistically significant. Continuous variables are represented by detailed sample descriptions with an average confidence interval of 95%. Categorical variables are expressed by counts and weighted percentages. The abnormal distribution is represented by median and Q1-Q3. The normal distribution is described by the median and standard deviation. Participants were divided into diabetic nephropathy group and non-diabetic nephropathy group according to whether ACR was greater than 30. In order to maximize the statistical efficiency and minimize the deviation, the missing data were interpolated for several times, and the sensitivity analysis of the interpolated data was analyzed to assess whether the generated data was significantly different from the original data. After sensitivity analysis, there was no statistically significant changes between the two groups. Therefore, tour interpolated data is consistent with the Robin guidelines research.

The statistical analysis of this study mainly consists of the following three stages to assess the correlation between GLB and diabetic nephropathy in selected subjects.

(1)The weighted single-factor and weighted multi-factor logical regression models were established, We established four different models according to the type of variables: model 1, without adjust any variables, that is, the single-factor logical regression model. Model 2 was adjusted for age, sex, race, BMI, waist. Model 3 was adjusted for variables in model 2 as well as GGT, BUN, Hba1c, ALT, AST, UA, TP, and ALB Model 4 was adjusted for variables in model 3 plus smoking, drinking status, HDL, TG, TBIL, and HBP.

(2)The smooth curve fitting graph was established and adjusted according to the covariables contained in model 4.The linear relationship between GLB and diabetic nephropathy was observed after logical regression.

(3) Subgroup analysis and weighted hierarchical logical regression were conducted on all subgroups to determine the stability of the results. Meanwhile, the GLB was converted into categorical variables based on the quartile for interactive testing. In addition, in the effect correction test, likelihood ratio test is carried out for the interaction terms between subgroups.

## Result

### Basic Information Characteristics When Obtaining Participant Data

**Figure 1:**
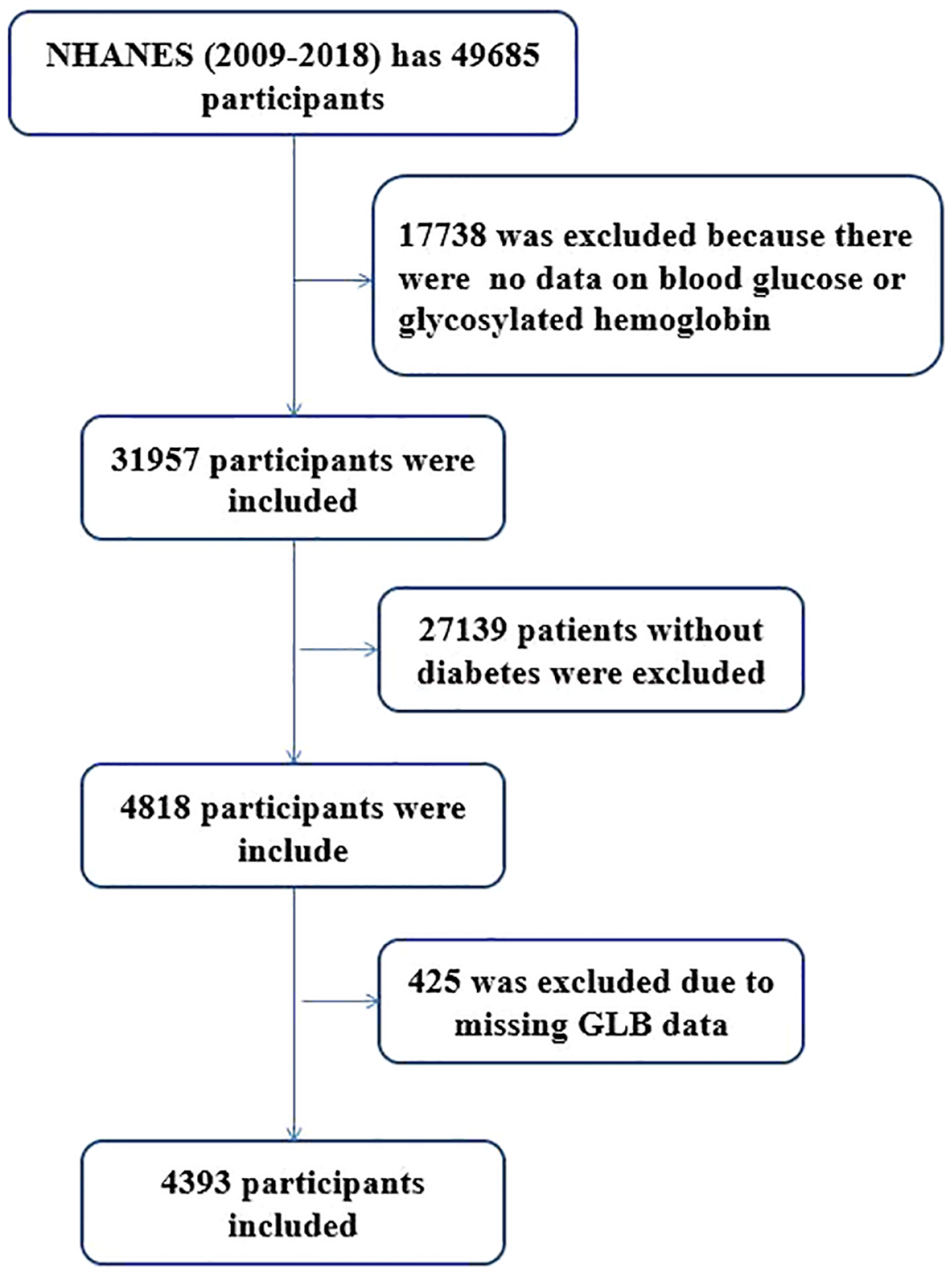
Flowchart of participant selection.

A total of 49,694 participants from the NHANES dataset were selected for this study, which lasted for ten years and five cycles. After screening according to the above strict criteria, a total of 4,393 diabetic patients with an average age of 60.4 ± 14.5 years were enrolledin the final analysis (**Figure 1**). 2,315 male patients (52.7%) were slightly higher than 2,078 females (47.3%), and the proportion of males (57.3%) was higher than that of females (42.7%). Diabetic nephropathy patients’ age (63.8 ± 13.4), waist circumference(110.1 ± 16.8 cm), glycosylated hemoglobin(7.2(6.3, 8.6) %), ALP (76.0(61.0, 95.0) g/L), BUN(6.1(4.6, 8.6 U/L) and GLB (31.0 (28.0, 35.0) g/L)were significantly higher than those of non-diabetic nephropathy.The proportion of participants with diabetic nephropathy varies by race. In contrast, HDL (1.2 (1.0, 1.4) mmol/L) and ALB (42.0, (39.0,44.0) g/L) were higher in non-diabetic nephropathy groups than in the diabetic nephropathy patients. There was no significant difference in BMI or alcohol consumption between the two groups (**Table 1**).

**Table 1:** Demographic characteristics describe whether diabetic nephropathy occurs.

Variables	Total (n = 4393)	Is there diabetic nephropathy?		*P*-value
		No (n = 3108)	Yes (n = 1285)	
Sex, n (%)				< 0.001
Male	2315 (52.7)	1579 (50.8)	736 (57.3)	
female	2078 (47.3)	1529 (49.2)	549 (42.7)
Age, Mean ± SD	60.4 ± 14.5	59.0 ± 14.8	63.8 ± 13.4	< 0.001
Race, n (%)				0.016
Mexican American	776 (17.7)	533 (17.1)	243 (18.9)	
Other Hispanic	478 (10.9)	354 (11.4)	124 (9.6)	
Non-Hispanic White	1514 (34.5)	1093 (35.2)	421 (32.8)	
Non-Hispanic Black	1020 (23.2)	688 (22.1)	332 (25.8)	
Other Race	605 (13.8)	440 (14.2)	165 (12.8)	
BMI(kg/m^2^), Mean ± SD	32.1 ± 7.6	32.0 ± 7.6	32.3 ± 7.7	0.349
Waist(cm), Mean ± SD	108.7 ± 16.7	108.2 ± 16.7	110.1 ± 16.8	< 0.001
Alchol use, Mean ± SD	1.5 ± 1.8	1.5 ± 2.0	1.5 ± 1.0	0.724
Smoking, n (%)				0.007
No	2250 (51.2)	1633 (52.5)	617 (48)	
Yes	2143 (48.8)	1475 (47.5)	668 (52)	
Hypertension, n (%)				< 0.001
No	829 (18.9)	691 (22.2)	138 (10.7)	
Yes	3564 (81.1)	2417 (77.8)	1147 (89.3)	
Hba1c(%), Median (IQR)	6.8 (6.0, 7.8)	6.6 (6.0, 7.6)	7.2 (6.3, 8.6)	< 0.001
ALB(g/L), Median (IQR)	41.0 (39.0, 43.0)	42.0 (39.0, 44.0)	40.0 (38.0, 43.0)	< 0.001
ALT(U/L), Median (IQR)	21.0 (16.0, 29.0)	22.0 (16.0, 30.0)	20.0 (15.0, 28.0)	< 0.001
HDL(mmol/L),Median (IQR)	1.2 (1.0, 1.4)	1.2 (1.0, 1.4)	1.1 (1.0, 1.4)	< 0.001
AST(U/L), Median (IQR)	22.0 (18.0, 28.0)	23.0 (19.0, 28.0)	22.0 (18.0, 28.0)	0.003
GLB(g/L), Median (IQR)	30.0 (27.0, 33.0)	29.0 (27.0, 32.0)	31.0 (28.0, 35.0)	< 0.001
ALP(U/L), Median (IQR)	72.0 (58.0, 90.0)	71.0 (58.0, 87.0)	76.0 (61.0, 95.0)	< 0.001
BUN(mmol/L),Median (IQR)	5.4 (3.9, 6.8)	5.0 (3.9, 6.4)	6.1 (4.6, 8.6)	< 0.001

BMI, Body Mass Index; ALB, Albumin; ALT, alanine aminotransfease; AST, aspartate transaminase; ALP, alkaline phosphatase; BUN, blood urea nitrogen.

### Univariate Analysis of Factors Related to Diabetic Nephropathy

Univariate logistic regression analysis (**Table2**) showed that gender, age, race, GGT, waist circumference, BUN, glycosylated hemoglobin, ALP, TP, UA, GLB, TG, smoking, HBP, HDL and ALB were the related factors of diabetic nephropathy. Women have a lower risk of diabetic nephropathy than men. Other Hispanics had a lower risk of diabetic nephropathy compared toMexican-Americans, and there was no statistical difference between other ethnic groups and Mexican-Americans. HDL and ALB were negatively correlated with the occurrence of diabetic nephropathy. On the contrary, some factors were positively correlated with the occurrence of diabetic nephropathy, including age, GGT, waist circumference, BUN, glycosylated hemoglobin, ALP, TP, UA, GLB, TG, smoking, and HBP, etc.

**Table 2:** Univariate analysis of association between factors of T2DM and diabetic nephropathy.

Variable	Diabetic nephropathy	
	OR (95%CI)	P-value
Gender		
Male	1	
female	0.77 (0.68~0.88)	<0.001
Age	1.03 (1.02~1.03)	<0.001
GGT	1 (1~1)	<0.001
RACE		
Mexican American	1	
Other Hispanic	0.77 (0.6~0.99)	0.043
Non-Hispanic White	0.84 (0.7~1.02)	0.08
Non-Hispanic Black	1.06 (0.87~1.29)	0.579
Other Race	0.82 (0.65~1.04)	0.103
BMI	1 (1~1.01)	0.349
Waist	1.01 (1~1.01)	<0.001
Alchol use		
No	1	
Yes	0.99 (0.95~1.04)	0.727
BUN	1.21 (1.19~1.24)	<0.001
Hba1c	1.28 (1.24~1.33)	<0.001
ALT	1 (1~1)	0.705
AST	1 (1~1)	0.957
ALP	1.01 (1~1.01)	<0.001
TP	1.03 (1.02~1.04)	<0.001
UA	1 (1~1)	<0.001
GLB	1.08 (1.06~1.09)	<0.001
TG	1.1 (1.05~1.14)	<0.001
Smoking		
no		
yes	1.2 (1.05~1.37)	0.006
HBP		
no		
yes	2.38 (1.95~2.89)	<0.001
HDL	0.74 (0.61~0.88)	0.001
ALB	0.91 (0.89~0.93)	<0.001
TBIL	0.99 (0.98~1)	0.147

BMI, Body Mass Index; ALB, Albumin; ALT, alanine aminotransfease; AST, aspartate transaminase; ALP, alkaline phosphatase; BUN, blood urea nitrogen; TBIL, total bilirubin; GLB, globulin.

### Multivariate Analysis of GLB and Related Factors of Diabetic Nephropathy

Four logical regression models were established to analyze the relationship between GLB and diabetic nephropathy. **Table 3** indicated the relationship between GLB and diabetic nephropathy in detail, and its effect value is expressed as OR and 95%CI. The magnitude of the effect value can be interpreted as a relative increase in the risk of diabetic nephropathy for each additional GLB unit. For example, in unadjusted model 1, the effect value was 1.08 (1.06-1.09) could be interpreted as an 8% increase in the risk of diabetic nephropathy for each additional GLB unit. In slightly adjusted model 2, the effect value was 1.08 (1.07-1.10), with an 8% increase in the risk of diabetic nephropathy for each additional GLB unit. In further adjusted model 3, the effect value was 1.10 (1.07 to 1.13) indicating a 10% increase in the risk of diabetic nephropathy for each additional unit of GLB. In fully adjusted model 4, the effect value was 1.10 (1.07-1.13), that is, each additional GLB unit increased the risk of diabetic nephropathy by 10%, P < 0.05, which was statistically significant. To verify the stability of the results, sensitivity analysis and subgroup analysis were conducted, and smooth fitting curves of GLB and diabetic nephropathy were plotted., The participants were divided into three groups according to GLB level and verified by four models. In all models, the group with the highest GLB content had the highest risk of developing diabetes, followed by the moderate group, with a consistent trend test, P < 0.001(**Table 3**). After fully adjusting its potential confounding factors based on the clinical consensus, GLB can be considered to have a strong positive correlation with the incidence of diabetic nephropathy if the influencing factors changed bymore than 10%.

**Table 3:** Multivariate analysis of association between GLB and diabetic nephropathy.

Variable	Model 1		Model 2		Model 3		Model 4	
	OR (95%CI)	P-value	OR (95%CI)	P-value	OR (95%CI)	P-value	OR (95%CI)	P-value
GLB	1.08 (1.06~1.09)	<0.001	1.08 (1.07~1.10)	<0.001	1.10 (1.07~1.13)	<0.001	1.10 (1.07~1.13)	<0.001
GLB group								
GLB low	1		1		1		1	
GLB middle	1.32 (1.11~1.57)	0.002	1.39 (1.16~1.66)	<0.001	1.26 (1.04~1.52)	0.016	1.26 (1.05~1.52)	0.015
GLB high	2.3 (1.94~2.72)	<0.001	2.51 (2.1~3)	<0.001	1.86 (1.53~2.26)	<0.001	1.88 (1.54~2.28)	<0.001
Trend test	1.54 (1.41~1.67)	<0.001	1.6 (1.47~1.75)	<0.001	1.37 (1.24~1.51)	<0.001	1.38 (1.25~1.52)	<0.001

Model 1: Non-adjusted.

Model 2: Age, gender, Race, BMI, waist.

Model 3: Model 2 + GGT, BUN, Hba1c, ALT, AST, UA, ALB.

Model 4: Model 3 + Smoking, Drinking, HDL, TG, TBIL.

### Subgroup Analysis and Curve Fitting

**Figure 2:**
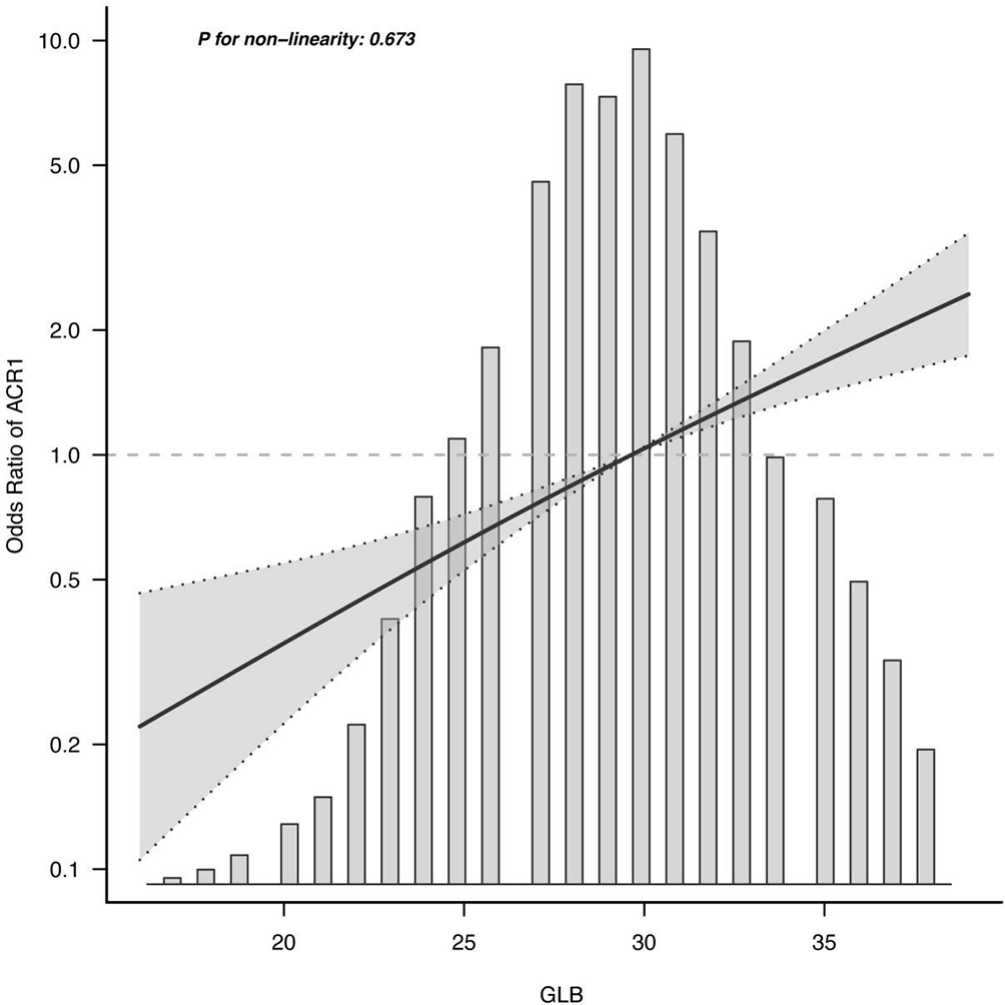
Curve fitting of serum globulin and diabetic nephropathy.

**Figure 3:**
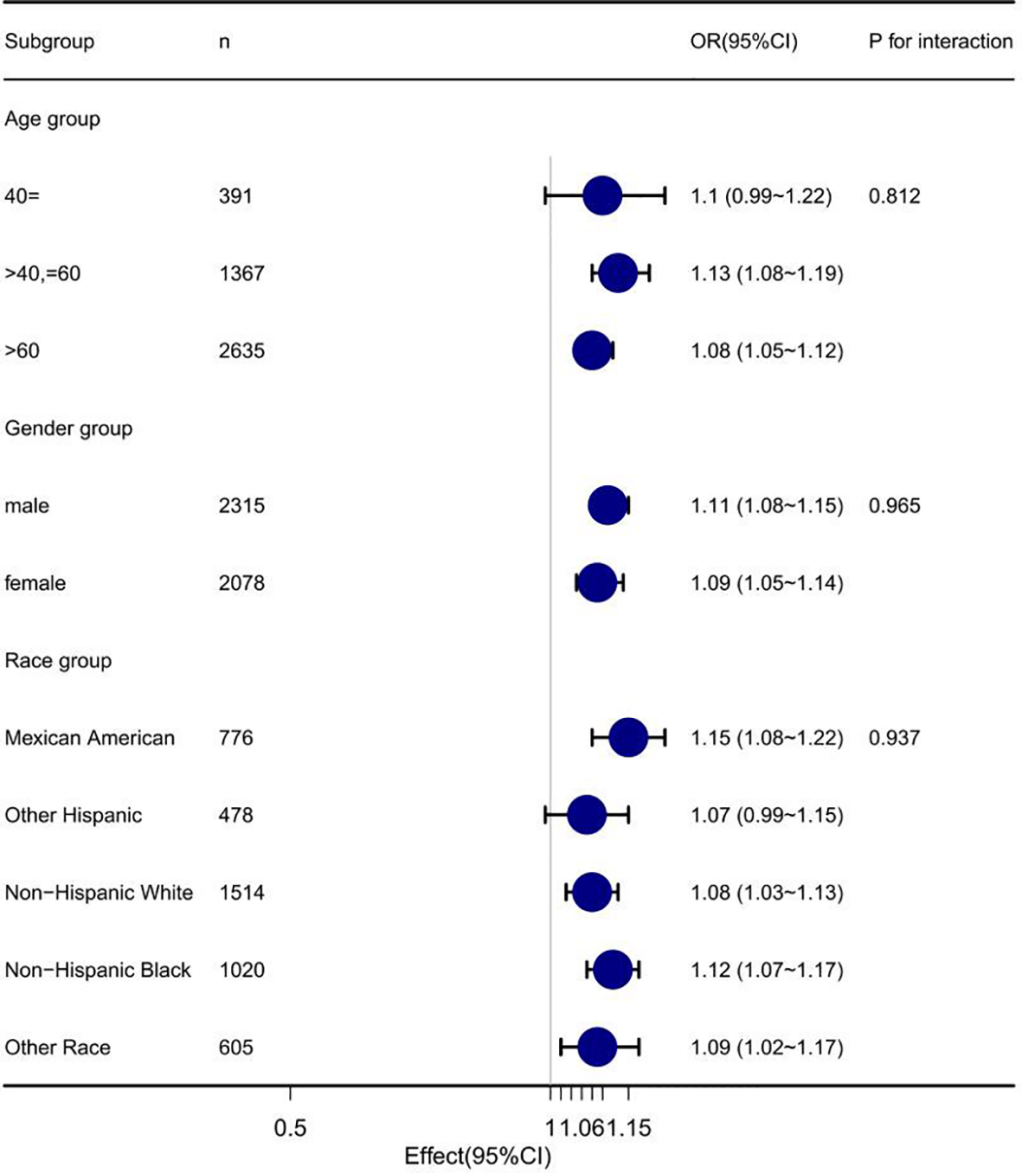
Forest plot of serum globulin and diabetic nephropathy.

After adjustment according to model 4, the fitting curve of GLB and diabetic nephropathy were drawn (**Figure 2**), in order to better explain the relationship between GLB and diabetic nephropathy. The results showed that there was a linear relationship between GLB and diabetic nephropathy (P for non-linearity = 0.673). The effects of different GLB levels were equal. In addition, we investigated whether there were differences in age, sex and race between GLB and diabetic nephropathy. The results revealed that the relationship between GLB and diabetic nephropathy was stable in all subgroups (**Figure 3**), there was no interaction (*P* > 0.05).

## Discussion

With the development of the world economy, the incidence of diabetes in the world has increased significantly. It is estimated that by 2040, the number of people with diabetes will reach 642 million, accounting for about 10% of the world’s total population (22). Diabetes is one of the most common chronic diseases confering about a two-fold excess risk for coronary heart disease, major stroke subtypes, and deaths attributed to other vascular causes (23). And patients tend to have pathological changes in other organs during a period of time (24), which will bring a lot of psychological and economic burden to patients and reduce their life experience (25). Therefore, a full understanding of the risk factors for these lesions can reduce the risk of diabetic complications and improve the well-being of patients.

This study investigated diabetic nephropathy, one of the most feared diabetic chronic microvascular complications of diabetes (26). The results suggested that high levels of GLB was positively correlated with the occurrence of diabetic nephropathy. To control for potential confounders, we established four logical regression models to analyze the association between GLB and diabetic nephropathy. In fully adjusted model 4, the effect value is 1.10 (1.07-1.13). This means that for each additional unit of GLB, the risk of diabetic nephropathy increases by 10%. According to the clinical consensus, after fully adjusting the potential confounders, the effect value of GLB has changed more than 10% of the influencing factors, which can be considered that there is a strong positive correlation between GLB and the incidence of diabetic nephropathy. Simultaneously, we divided GLB into three groups and conducted sensitivity analysis on the results, which showed that the results were stable and reliable (**Table 2**). Additionally, we validated the results in age, sex and race subgroups and found that they were stable in all subgroups without interaction (**Figure 3**). The fitting curve (**Figure 2**) between GLB and diabetic nephropathy was drawn after adjustment according to model 4, in order to better present this result and observe the linear relationship between GLB and diabetic nephropathy. This is also consistent with our validation of trend lines.

Some studies may partially explain the underlying mechanisms of GLB and diabetic nephropathy. GLB is one of the common inflammatory factors and has been used in liver function analysis. Liu et al. found that GLB elevation may reduce BCL2 expression through TNF regulation (22). Khater et al. found in the animal experiment of diabetic nephropathy, that the expression of BCL2 could effectively reduce renal tissue damage in diabetic nephropathy rats. Animal studies have also indicated that GLB aggravates renal injury by promoting the expression of TNF- α, IL-6 and IL-1 β (27).

Furthermore, Guo et al. proposed that miRNA-29c can regulate the expression of inflammatory cytokines in diabetic nephropathy by targeting tritetraproline (28). GLB can inhibit some miRNA expression by stimulating the secretion of inflammatory cells (29). RS et al. found a positively correlation between GLB and the occurrence and development of diabetes in a cohort study of Indians (30). Nakazawa D et al. found that elevated GLB can stimulate the secretion of neutrophils (31), which is also a risk factor for diabetic complications (32). These studies also indicated the association between GLB and diabetic nephropathy to some extent. Therefore, we speculate that lowering GLB level may be a good way to regulate the health status of diabetics patients. However, more prospective studies are needed to determine whether this study is applicable to a wider population.

### Strength of the Study

In this study, compared with previous studies, some other aspects are worth mentioning. The population we selected was more broadly representative, due to the polycentric nature of the NHAENS database (12). Furthermore, in order to more intuitively represented the relationship between GLB and diabetic nephropathy, a smooth fitting curve was drawn to illustrate the relationship.

### Limitations of the Study

Unfortunately, there are some limitations to this study. Although our results suggest that GLB level was strongly positively correlated with the occurrence of diabetic nephropathy, this study was a cross-sectional study, it is impossible to draw a causal relationship between the two, which require further cohort studies or case-control studies to clarify the relationship between GLB levels and diabetic nephropathy. Secondly, we excluded people younger than the population we included in the study, but considering that we used the population-weighted weight of the official NHANES website, this disadvantages has been well avoided. Moreover, our population inclusion was limited by the NHANES database only including some normal populations, excluding other countries and some special populations (e.g., pregnant women, cancer patients, etc.). Whether the relationship between GLB and diabetic nephropathy applies to this population is unclear.

## Conclusion

In conclusion, the results of this cross-sectional study revealed that GLB was significantly positively correlated with the occurrence of diabetic nephropathy in diabetic patients. However, the specific mechanism and whether it is applicable to other populations need to be further studied. This study provides a new perspective for exploring the pathogenic factors of diabetic nephropathy.

## Data Availability Statement

Publicly available datasets were analyzed in this study. This data can be found here: All data are available on the NHANES website.

## Author Contributions

XH conceived the idea; JW and FL wrote the manuscript; RK collected and read the literature and revised the article; XH read through and corrected the manuscript. All authors read and approved the final manuscript. XH is corresponding author of this paper.

## Conflict of Interest

The authors declare that the research was conducted in the absence of any commercial or financial relationships that could be construed as a potential conflict of interest.

## Publisher’s Note

All claims expressed in this article are solely those of the authors and do not necessarily represent those of their affiliated organizations, or those of the publisher, the editors and the reviewers. Any product that may be evaluated in this article, or claim that may be made by its manufacturer, is not guaranteed or endorsed by the publisher.

## Abbreviations

GLB, globulin; DN, diabetic nephropathy; NHANES, the National Health and Nutrition Examination Survey; GLP, globulin; ACR, urinary microalbumin creatinine ratio; BMI, body mass index; GGT, gamma-glutamyltransferase; BUN, blood urea nitrogen; ALP, alkaline phosphatase; TP, total protein; UA, uric acid; TG, triglyceride; HBP, high blood pressure; HDL, high-density lipoprotein; ALB, albumin.
